# “All Children in Focus”: Effects of a Universal Parenting Program at a 6-Month Follow-Up in a Randomized Controlled Trial in Sweden

**DOI:** 10.1007/s11121-024-01681-y

**Published:** 2024-05-15

**Authors:** Isabella Victoria Cinà, Lene Lindberg, Pia Enebrink

**Affiliations:** 1 Department of Global Public Health, Karolinska Institutet, Stockholm, Sweden; 2 Center for Epidemiology and Community Medicine Region Stockholm, Box 45436, S-104 31 Stockholm, Sweden; 3 Division of Psychology, Department of Clinical Neuroscience, Karolinska Institutet, Stockholm, Sweden

**Keywords:** Health promotion, Universal parenting program, ACF program, Child wellbeing, Parenting, Randomized controlled trial

## Abstract

Parenting programs have been shown to empower parents and prevent mental health problems in children. However, few programs are designed to promote wellbeing, which led to the development of the Swedish-promotive universal program, “All Children in Focus” (ACF). This study aimed to understand the effects of parents’ participation in the ACF program on parents’ emotion regulation and parenting practices over a 6-month follow-up and if intervention-produced changes predict child wellbeing (CW). Parental self-efficacy (PSE) was also included to assess the relation with parental outcomes and CW. This is an evaluation of a multicenter randomized waitlisted control trial conducted in Stockholm County, Sweden, with parents of children aged 3 to 12 years (*n* = 621) in 2012. Parents were randomized 1:1 to enter the program or to a waitlist. Questionnaires were completed by parents at baseline, post-intervention, and 6 months post-baseline. We used generalized mixed modeling to test effects on parental outcomes over time and regression analyses to study the predictive role of parental outcomes on CW. From baseline to the post- and to the 6-month follow-ups, parents in the intervention group reported greater levels of emotion regulation skills, cognitive reappraisal, and positive parenting than did parents in the control group. A decrease in negative and harsh parenting was evident in both groups, although it was greater in the intervention group. An increase in PSE was associated with high negative parenting at baseline in the intervention group. Child gender moderated positive parenting, suggesting that parents of girls in the intervention group had more favorable development of positive strategies. The findings indicate that a change in PSE predicts a change in CW at 6 months. The ACF program can be used to strengthen and develop parenting. This study advances our knowledge about the potential advantages of using a health-promoting approach to strengthen the wellbeing of families in the general population. Clinical trial registration: ISRCTN70202532 Current Controlled Trials

**Supplementary Information:**

The online version contains supplementary material available at 10.1007/s11121-024-01681-y.

## Background

Childhood is a critical period for cognitive, emotional, and social development (Halfon & Forrest, 2018). Experiences early in life may have a cumulative effect on health and wellbeing across the lifespan (Daines et al., 2021). Attention to child development through proactive preventative action and health promotion is therefore a crucial public health investment, not only to improve a child’s health status but also for future outcomes in adulthood. The association between parenting and child development is well documented (Thomas Boyce & Hertzman, 2018). Although parenting is shaped by sociocultural-historical contexts, parenting is a universal experience and is known to be both a powerful protective factor and a risk factor for the physical, emotional, and social development of a child from infancy to adulthood (Stewart-Brown, 2008).

### Parenting Programs

The mediating role of the family environment on a child’s development has been supported by years of research and has led to the development of a range of parenting interventions designed to increase parents’ knowledge and skills, improve parenting practices, and manage parental stress to mitigate child behavioral and emotional problems (Leslie et al., 2016). Three different intervention models are described: (a) indicated, for parents with children having early signs of problems where the intervention intends to prevent the onset of more severe problems; (b) selective, for subgroups of parents with risk factors that may increase the risk for certain outcomes; and (c) universal, which is open to all parents in the general population (Leslie et al., 2016; Sanders & Kirby, 2014). Universal parenting programs (UPPs) aspire to prevent risk factors for future problems or to promote positive health (Leslie et al., 2016; Salari & Enebrink, 2018). Research on UPPs aimed at preventing child problems has significantly increased in the last 20 years; however, programs developed to promote child wellbeing are limited.

The efficacy and effectiveness of selective and indicated parenting programs within various parent and child populations have been described (Barlow & Coren, 2018; Hudson et al., 2023). UPPs are not widely evaluated, but there is promising evidence for their effectiveness and cost-effectiveness. An example of an internationally implemented parenting program with UPP as one level of intervention is the Triple P-Positive Parenting Program (Sanders et al., 2014). The UPP version of the Triple P has been shown to improve children’s social, emotional, and behavioral outcomes as well as various parenting outcomes, such as parenting practices (Sanders et al., 2014). Triple P has demonstrated long-term effectiveness, with some studies suggesting that it has maintained intervention effects on reducing child externalizing behavior and improving wellbeing later in adolescence (Kim et al., 2018). However, other studies of Triple P found no significant long-term effects on child externalizing behavior (Eisner et al., 2012; Sampaio et al., 2015), parenting practices (Eisner et al., 2012), or parental mental health (Sampaio et al., 2015). A randomized controlled trial (RCT) of the Incredible Years program delivered universally found that participating parents indicated reduced harsh parenting practices, child behavior problems, and increased positive parenting and parental self-efficacy (Reedtz et al., 2011). The effects on parenting and self-efficacy were maintained at the 1-year follow-up; however, the effect on children’s behavioral problems was not maintained. Evaluations of Tuning in to Kids have shown to improve emotional regulation strategies in parents of preschool aged children (Havighurst et al., 2010). Nevertheless, not all UPP evaluations found significant evidence for effects on parent and child outcomes (Simkiss et al., 2013). Participants’ socioeconomic status is rarely included in such studies, but Hahlweg et al. (2010) found higher participation from parents from higher socioeconomic areas which opposes the idea of adopting a health promoting approach to enhance families’ health and wellbeing. Another finding by Hahlweg et al. (2010) is that no differences were found in program effects on parenting and child behavior outcomes in single parent mothers contrary to two-parent households. Based on the mixed results, more studies are required to obtain strong evidence on the long-term effects of UPPs in promoting health in the general population.

### “All Children in Focus” Program

“All Children in Focus” (ACF) is a UPP for parents whose children are 3 to 12 years old that was developed in 2012 in Stockholm County, Sweden. The program was part of a Swedish national governmental initiative of UPPs (Swedish Ministry of Health and Social Affairs, 2009). The literature on evidence-based parenting programs’ risk and protective factors was reviewed and considered in ACF’s development, as were interviews about relevant components with parents and a reference group of professionals working with parenting support (unpublished data). This led to the development of a 4-session group-based program that focused on strategies for coping with various parenting situations, such as everyday stressors and emotion regulation difficulties.

fSupplTheoretically, the program is based on social learning theory and includes a focus on the importance of a positive child–parent relationship, such as giving positive attention to what works, reinforcing positive child behavior, setting clear and healthy boundaries when needed, and discussing how to be a role model as a parent and how to regulate stress and anger. It is also influenced by attachment theory, specifically in emphasizing the importance of a sensitive and attuned child relationship, which merges well with the positive parent–child relationship focus in social learning theory and in how both of these approaches highlight child-directed play as a way to accomplish this. The ACF considers influences on family functioning by addressing parental stresses and experiences (Lindberg et al., 2013). Some similarities are drawn to other UPPs, such as Triple P (Sanders & Kirby, 2014), the International Child Development Program (Solheim Skar et al., 2015), and the Family-Links Nurturing Program (Simkiss et al., 2013), such as promoting child wellbeing, parental empowerment, and enhancing parental attention and empathy. Similarities in strategies and formats may also be seen in relation to targeted parenting programs used in Sweden, such as the Comet, Cope, Incredible Years, and the attachment-focused Connect program (Stattin et al., 2015). However, these have a larger number of sessions and include problem-focused program content or individual meetings that are not in line with the health-promoting focus of the ACF program.

An ACF pilot study with a pre-post measurement design revealed that the program significantly improved parenting strategies, parental self-efficacy, and child wellbeing (CW) (Enebrink et al., 2015). Most effects were maintained after 4 months, with parental university education as a moderator of CW. These promising findings led Lindberg and colleagues to run a randomized waitlist-control trial of the ACF program to assess intervention effectiveness on parental self-efficacy and parent-rated CW and possible moderating variables over a 6-month follow-up period (Lindberg et al., 2013; Ulfsdotter et al, 2014). The results indicated an interaction effect on both primary outcomes, corresponding to a moderate increase in the effect size of the intervention group on parent-rated self-efficacy and CW compared with that of the control group. A moderating effect was found in that having a higher level of parental education and having more than one child increased parental self-efficacy. Furthermore, having older children was beneficial for increasing CW, whereas poor parental mental wellbeing moderated both parental self-efficacy and CW. The ACF trial revealed no moderating effects of country of birth, which supports the guiding principles of positive parenting as cross-culturally robust (Sanders, 2008; Ulfsdotter et al., 2014). A cost-effectiveness analysis of the program showed positive findings (Ulfsdotter et al., 2015); however, 6 months is a short analysis period, and the cost-effectiveness calculation warrants further research.

### Study Aim

The aim of the present study was to increase the knowledge of how the Swedish health-promoting universally delivered ACF program affected secondary outcomes in an earlier published RCT study (Ulfsdotter et al, 2014), i.e., parenting practices and parental emotional regulation, and how these parenting components are related to child wellbeing (CW). We also considered how parental outcomes and CW were related to the primary outcome of parental self-efficacy (PSE) in the ACF. In line with previous UPP evaluations, we hypothesized that parents who participate in ACF would report an increase in emotion regulation and positive parenting practices over time compared to those in the control group (Havighurst et al., 2010; Reedtz et al., 2011; Sanders et al., 2014). We also hypothesized that intervention-produced changes in parental emotional regulation and parenting practices would be associated with changes in parents’ perceptions of their child’s wellbeing over the follow-up period since parenting strategies have been associated with child outcomes (Stewart-Brown, 2008). Finally, based on earlier research, we examined possible moderators of parenting practices and parental emotional regulation.

## Method

### Study Design

This study used secondary outcome data collected from a multicenter randomized waitlist-controlled trial of the ACF program (Lindberg et al., 2013). Parents were randomly allocated to either participate in the universally delivered ACF program (*n* = 323) or be placed on a waitlist to enter the program 6 months later (*n* = 298). The current study retained an RCT study design and used data collected at three time points: baseline pre-intervention, 2 weeks post-intervention, and 6 months after baseline.

### Setting and Population

The ACF program was put into practice in 2012 across 11 city districts and municipalities in Stockholm County, Sweden. Parents of children aged 3 to 12 years living within these areas were included in the study. The city districts and municipalities were chosen so that a representative sample of families would be covered with respect to household income, ethnicity, and education level. The program was delivered at the local level in schools, family centers, and other community centers.

### Procedures

#### Recruitment

Recruitment took place locally and was headed by a locally assigned coordinator together with the research team. Examples of the recruitment methods used were direct contact with families and via mailed letters and advertisement in local establishments such as supermarkets, schools, preschools, and child health centers. A promotional film about the ACF program was also produced and distributed locally to recruit participants. Parents showing interest were invited to local information meetings about the ACF and the study. Parents entered the study after providing signed informed consent.

#### Randomization and Participation

The researchers of the primary ACF trial performed randomization after collecting baseline data from the participants during spring and autumn 2012. In total, 621 participants were randomized at the individual level to the intervention or control group at a 1:1 ratio. This was performed in IBM SPSS Statistics (version 20) using the random-sampling function. To avoid the risk of contamination in the intervention group, parents or caretakers of one child were randomized as one unit, resulting in 323 parental units (75.1% mothers and 24.9% fathers) assigned to the intervention group and 298 to the control group (71.3% mothers and 28.7% fathers). The CONSORT flow chart published by Ulfsdotter et al. (2014) provides a detailed overview of the enrollment of participants. In the ACF study, 39 groups were administered, with each group targeting parents of children aged 3 to 12 years. In 31 of the 39 groups, the age range for the children varied from 5 up to 9 years.

The sample size for statistical power analyses is presented in the study protocol and suggest that a sample of 220 participants per condition would be needed to detect an effect size of 0.4 at 90% power (*p* < 0.05) with an intraclass correlations estimate of 0.1. Allowing for a 6-month loss to follow-up of approximately 20%, the researchers aimed for the enrollment of at least 300 participants per group. In this study, data from a total of 613 participants—317 (238 mothers and 79 fathers) in the intervention group and 296 (211 mothers and 85 fathers) in the control group—were utilized.

Post-measurement attrition rates were 8.2% and 18.9% at the 6-month follow-up, indicating that the sample used in this study met the power and sample size requirements of the study protocol. Post-intervention questionnaires were sent out when group leaders reported that they had finished the intervention, and follow-up questionnaires were sent out 6 months after baseline. Reminding by phone or text was performed after 2 weeks for each time point. The mean time to response post-intervention was 1.27 weeks (SD = 1.27; range = 0–5 weeks) for the intervention group and 1.18 weeks (SD = 1.00; range = 0–5 weeks) for the control group. The mean time to response after the 6-month follow-up was 1.21 weeks (SD = 0.95; range = 0–8 weeks) for the intervention group and 1.18 weeks (SD = 1.12; range = 0–8 weeks) for the control group.

### ACF Intervention

ACF is a group-based UPP with four structured sessions delivered biweekly covering evidence-based components: positive attention and warmth, parent–child time and child-directed play, positive parenting strategies, and consistent parenting (Ulfsdotter et al, 2014). The duration of each session is 2.5 h, and a new theme is introduced each session through group discussions, role-playing, and films. The first session is titled “showing love” (positive attention and warmth) and includes reflections on how to show empathy and positive attentiveness in the interaction with the child and how to express positive reactions to and strengthen the child’s positive behaviors. The theme for the second session, “being present” (parent–child time and child-directed play), targets how to be sensitive to the child when together or in child-directed play. During the third session, “showing the way” (positive parenting strategies), parents discuss and are taught strategies on how to cope with parental stress and anger and with child behaviors. The last session, “pick your battles” (consistent parenting), includes limit setting and strategies for coping with conflicts. Examples of parent–child interactions and role-plays during the sessions are adapted to the ages of the participating parents’ children. Parents are encouraged to try the content at home between the sessions. After completion of the program, parents are offered one booster session after 2 to 3 months; however, in the ACF study, it was offered after the 6-month follow-up. Parents can choose to participate in a booster session about either siblings, boys and girls, or teenagers. To provide further information to parents depending on development and different ages, a web page is provided where the material is written by researchers with expertise on topics such as bullying, language development, homework, and collaboration with the school.

Group leaders received 4.5 days of training on how to deliver the ACF program. Each group has two group leaders. During the ACF study, group leaders filled out checklists after each session with questions about how the session was implemented and received supervision on video-recorded group sessions.

### Measures

The questionnaires were administered to parents in person to measure parental emotion regulation, parenting practices, and CW. Demographic characteristics and parents’ mental health symptoms (measured with the General Health Questionnaire—GHQ-12 (Goldberg & Williams, 1988)) were collected at baseline. Each of the 12 items in the GHQ were scored on a 4-point Likert scale with a total sum, where a higher score indicates more symptoms.

Parental emotion regulation refers to the capacity to adjust and control how affect is practiced and articulated and was assessed by the Swedish version of the Emotion Regulation Questionnaire (ERQ) (Enebrink et al., 2013; Gross & John, 2003). The questionnaire covers 10 self-reported items that measure the use of two emotional regulation strategies through the following subscales: cognitive reappraisal (6 items) and expressive suppression (4 items). In this study, the two subscales were analyzed separately. The two ERQ subscales demonstrated satisfactory reliability in a sample of Swedish parents with children aged 10 to 13 years (Enebrink et al., 2013). The items were assessed on a 7-point Likert scale ranging from 1 (disagree) to 7 (totally agree) and then combined into a total mean score. A higher score indicates that emotional regulation strategies were applied more often. In this study, the Cronbach’s alphas were 0.81 and 0.85 for the reappraisal scale and 0.72 and 0.74 for the suppression scale across the repeated measures, indicating acceptable internal consistency.

Parenting practices reflect the approaches (i.e., child monitoring) and ways (i.e., harsh style) that parents practice their child rearing and were assessed by the self-reported Parenting Practices Interview (PPI) questionnaire modified from the Oregon Social Learning Centre’s Discipline Questionnaire and adjusted for parents of young children (Enebrink et al., 2013; Webster-Stratton et al., 2001). In this study, the two PPI subscales, harsh and inconsistent discipline (15 items) and praise and positive incentives (11 items), were applied and analyzed independently. The items were assessed on a 7-point Likert scale ranging from 1 (never/not at all likely/strongly disagree) to 7 (always/extremely likely/strongly agree), except for one item that was measured on a scale ranging from 1 (never) to 6 (6–7 times). A total sum score was then computed for each subscale, where a higher score implies more frequent use of these parenting strategies. The Cronbach’s alpha was between 0.81 and 0.82 for the harsh scale and 0.73 and 0.75 for the praise scale across the repeated measures, indicating acceptable internal consistency.

Parental self-efficacy (PSE (Bloomfield & Kendall, 2012)) was assessed with 48 items rated on a Likert scale from 0 to 10 and included questions about parental experiences of positive emotions, being with and guiding the child, empathy, rules, pressures from others, and acceptance of parenting. The total sum of the items ranges from 0 to 480, with a higher score indicating higher PSE. Cronbach’s alpha for the total PSE scale was 0.94 and 0.92.

Child wellbeing was measured using the 35-item parent-reported Child Wellbeing (CW) questionnaire. It was adapted from the KIDSCREEN questionnaire for use in parents with young children and measures parents’ perception of their child’s wellbeing (Lindberg et al., 2013, Ravens-Sieberer et al, 2014). Parents rated child physical activity and mental health, emotional development and independence, family relations, and social competence on a 5-point Likert scale. A total score was then computed where a higher score indicates better CW. In this trial, the Cronbach’s alpha was 0.93 at baseline and 0.93 at post- and follow-up measurement, suggesting very good internal consistency.

### Statistical Analysis

For all the statistical analyses, IBM SPSS Statistics (version 27) was used. An alpha of *p* < 0.05 (95% confidence interval) was used as the significance level, and an intent-to-treat (ITT) approach was applied in the statistical tests according to the original group allocations. Study completer and analyses per protocol (participation in at least one AFC session) were performed as a strategy to verify the findings. The participants’ baseline characteristics were explored with descriptive statistics, and differences between the intervention and control groups were analyzed with chi-square tests and *t*-tests.

To examine the effect of treatment (intervention and control groups) across three points of time points (pre-intervention, post-intervention, and 6 months post-baseline) on the outcomes of parental emotional regulation and parenting practices, generalized linear mixed modeling (GLMM) with repeated measures was used. Using GLMM is considered useful because it concurrently establishes between-group (treatment group) and within-group (time) variance and includes all participants in the analysis irrespective of having all or partial data across repeated measures (Jiang, 2007). Moreover, the covariant structure and distribution can be modified to better mirror the nature of the data. In this study, all GLMM analyses used first-order autoregressive models with homogeneous variance. A random intercept for individual participants and a normal or gamma distribution with identity or log links were specified in accordance with the outcome variable. Findings for variables with gamma distributions were analyzed with additional repeated ANOVA, Mann–Whitney *U* tests, and Wilcoxon signed-rank tests. Sequential Sidak correction was applied to control familywise error, and to determine the effect sizes, Cohen’s *d* was calculated, where 0.20–0.49 was considered a small effect size, 0.50–0.79 was considered moderate, and >0.80 was considered large. PSE was also included in the GLMM analyses to examine associations with the secondary parental outcomes. For this purpose, the change in PSE from baseline to post-intervention was measured. A change greater than 1 was coded as an increase, and a change less than 1 was coded as no increase. To evaluate the potential moderators of child age and gender, relationship status, parents’ education, and mental health symptoms, these variables were also included as continuous (child age) or dichotomized variables in the final step of the GLMM analyses. The GHQ-12 was used as a measure of mental health symptoms and was dichotomized with a cutoff of 11/12 for no or few symptoms versus the presence of symptoms. Study completer and analyses per protocol without imputation were conducted with repeated-measures ANOVA, including (1) all participants who answered questionnaires, i.e., also those randomized to the AFC but who did not participate, and (2) parents exposed to at least one ACF session. Next, to explore whether changes in parental outcome measures during the intervention period predicted changes in CW over the 6-month follow-up, a linear regression analysis was performed, also with PSE included.

## Results

### Attrition and Participation

Baseline data showed no differences between the intervention and control groups (see Table 1). The post-intervention measurements were completed by 563 out of 613 participating parents (91.8%), and the measurements at the 6-month follow-up were completed by 497 parents (81.1%). The attrition rate was significantly greater for the intervention group than for the control group at both post-intervention (${\chi }^{2}$ [1] = 4.45, *p* < 0.05) and 6-month follow-up (${\chi }^{2}$ [1] = 4.27, *p <* 0.05). Compared to the participants who completed the post-measurement, parents who did not complete this measurement were younger (*t*[611] = −2.10, *p* < 0.05), had lower average monthly incomes (*t*[583] = −3.68, *p* < 0.001), had younger children (*t*[611] = −2.31, *p* < 0.05), were born outside Sweden (${\chi }^{2}$ [1] = 8.67, *p* < 0.01), and had completed a lower level of education (${\chi }^{2}$ [1] = 4.60, *p* < 0.05). Compared with the participants who completed the 6-month follow-up, parents who did not complete this measure were more likely to be born outside of Sweden (${\chi }^{2}$ [1] = 21.85, *p* < 0.001), but there were no significant differences in other baseline characteristics. For the post- and follow-up measurements in the intervention group, no significant differences were detected in the ERQ or PPI scores between those who completed the intervention and those who dropped out.

**Table 1:** Parent-reported baseline characteristics of the participating parents and children, reported as the mean (standard deviation) or number (percent)

**Variables**	**Intervention (*n* = 317)**	**Control (*n* = 296)**	**Statistic**	***p* value**
Mother	238 (75.1)	211 (71.3)	*χ*^2^(1) = 1.13	0.289
Parental age (years)	38.09 (5.5)	38.38 (5.4)	*t*(611) = 0.65	0.928
Born in Sweden	249 (78.5)	222 (75.0)	*χ*^2^(1) = 1.08	0.298
Single parent	32 (10.1)	28 (9.5)	*χ*^2^(1) = 0.08	0.782
Number of children	2.13 (.9)	2.14 (.8)	*t*(611) = 0.15	0.884
University education	206 (66.0)	192 (64.9)	*χ*^2^(1) = 0.02	0.898
Household income^a^	55,108.3 (22,480.9)	58,154.2 (25,323.1)	*t*(583) = 1.54	0.124
Girl	135 (42.6)	128 (43.2)	*χ*^2^(1) = 0.017	0.896
Child age (years)	6.09 (2.6)	6.26 (2.6)	*t*(611) = 0.79	0.432

^a^Household income in Swedish krona (SEK, 1 Euro = 10.24 SEK [May 2021])

### Program Effects on Parent Outcomes

To specify the GLMM for each outcome variable, we initially conducted exploratory analyses. A normal distribution with an identity link was used for the ERQ and positive PPI subscales. A gamma distribution with a log link was used for the negative PPI subscale. The mean and standard error at each time point by treatment group and their interaction effects (group × time) are presented in Table 2.

**Table 2:** Mean score (standard error) at each measurement time point with GLMM-based repeated measures group × time interaction effect of the outcome measures by intervention (*n* = 317) and control (*n* = 295) group

**Outcome measures**	**T1**^a^	**T2**^b^	**T3**^c^	**GLMM**	***t*-tests**		
	***M (SE)***	***M (SE)***	***M (SE)***		**T1–T2**	**T2–T3**	**T1–T3**
**ERQ. Cognitive reappraisal**^d^							
Intervention	4.47 (0.06)	4.90 (0.06)	4.76 (0.06)	*F*(2,1657) = 12.07, *p* < 0.001	−7.76***	2.26*	−4.64***
Control	4.60 (0.06)	4.65 (0.06)	4.60 (0.06)		−0.86	0.85	0.02
**ERQ. Expressive suppression**							
Intervention	2.82 (0.07)	2.87 (0.07)	2.93 (0.07)	*F*(2,1659) = 1.07, *p* = 0.343	−1.00	−0.90	−1.72
Control	2.80 (0.07)	2.96 (0.07)	2.91 (0.07)		−2.71*	0.82	−1.65
**PPI. Harsh and inconsistent discipline**^e^							
Intervention	43.36 (0.55)	40.58 (0.53)	40.92 (0.55)	*F*(2,1610) = 4.37, *p* = 0.013	6.55***	−0.80	5.04***
Control	43.76 (0.57)	42.70 (0.57)	42.15 (0.58)		2.44*	1.24	3.27**
**PPI. Praise and positive incentives**							
Intervention	41.46 (0.48)	42.32 (0.50)	42.65 (0.51)	*F*(2,1624) = 4.18, *p* = 0.015	−0.87*	0.86	2.97**
Control	42.04 (0.50)	41.61 (0.50)	41.83 (0.52)		1.20	0.53	0.59

^*^p < 0.05; **p < 0.01; ***p < 0.001 (95% confidence interval)

^a^T1 = pre-intervention

^b^T2 = 2 weeks post-intervention

^c^T3 = 6 months post-baseline

^d^ERQ = Emotion Regulation Questionnaire

^e^PPI = Parenting Practices Interview

At baseline, we found no significant differences between the intervention and control groups across any of the outcome measures. For the cognitive reappraisal ERQ subscale, there was a significant interaction effect over 6 months (see Table 2). In the intervention group, parental cognitive reappraisal increased significantly from baseline to the 6-month follow-up, as compared to the control group. There was a significant increase from pre-intervention (T1) to post-intervention (T2) for the intervention group (*d* = 0.41), followed by a significant but small decrease at the 6-month follow-up (T3) (pre-f.u, *d* = 0.28). The control group showed no significant differences across the measurement period. Between-group effect sizes were small (*d* = 0.25 and 0.17, respectively). For the expressive suppression ERQ subscale, we found no interaction effect over the 6-month follow-up. The repeated measures between the groups and within the intervention group showed no significant differences (all *d*’s < 0.10). However, from T1 to T2, we found a significant increase in the control group. For the harsh and inconsistent discipline PPI subscale, we found a significant interaction effect (see Table 2). Reports of using negative parenting strategies decreased significantly in both groups over the follow-up period, but the intervention group showed a larger mean score decrease (pre-post *d* = 0.28; pre-f.u *d* = 0.25; between-group post *d* = 0.23, f.u *d* = 0.14). Analysis of the PPI subscale for praise and positive incentives demonstrated a significant interaction effect, indicating that the use of positive parenting practices increased more in the intervention group than in the control group over time (see Table 2). In the intervention group, the use of positive parenting increased significantly from T1 to T2 (pre-post *d* = 0.10), with a nonsignificant change during the follow-up period (pre-f.u *d* = 14). No significant differences between the groups were found at any of the time points (between-group *d* < 0.10). When an increase in PSE was added to the interaction with group and time (see Supplemental Table 1), there was no significant finding for expressive suppression of ERQ, while a significant finding was found for cognitive reappraisal of ERQ. The findings indicated that an increase in parental self-efficacy in the control group from T1 to T2 also contributed to an increase in cognitive reappraisal compared to no increase in parental self-efficacy (*t*(1625) = 2.47, *p* < 0.05). For the harsh and inconsistent discipline PPI, we found a significant interaction effect with PSE added to group and time (supplementary Table 2). An increase in parental self-efficacy from T1 to T2 appeared to be related to more negative parenting practices at baseline in the intervention group compared to parents with lower negative practices (*t*(1580) = 2.72, *p* < 0.01). The addition of PSE to the interaction of time and group in the case of praise and positive incentives for PPI indicated a tendency toward significance. Further comparisons showed that an increase in parental self-efficacy was related to more positive parenting practices in the intervention group from baseline (*t*(1594) = 2.61, *p* < 0.01) and during the follow-up (T1 (*t*(1594) = 3.77, *p* < 0.001), T2 (*t*(1594) = 2.93, *p* < 0.01)) than to no increase in parental self-efficacy.

Regarding moderators, we found no significant effects for child age, parental education level, parental mental health, or relationship status. A significant moderating effect was found for child gender and praise and positive incentives for PPI (*F*[2,1617] = 4.41, *p* < 0.05). Parents of girls in the intervention group showed greater increases in praise and positive incentives than parents of girls in the comparison group.

We also conducted study completer analyses and analyses per protocol using ANOVA repeated measures over the 3 measurement points (see Supplemental Tables). The results were in line with the earlier reported findings in Table 2. For completers, there was an interaction effect for the cognitive reappraisal ERQ subscale (Supplementary Table 2) over the 6-month period. No significant interaction effect was found for the expressive suppression ERQ subscale (Supplementary Table 2). For the harsh and inconsistent discipline PPI subscale and for the praise and positive incentives PPI subscale, significant interaction effects were found (Supplementary Table 2). According to the analyses per protocol, the interaction effect was significant for the cognitive reappraisal ERQ subscale (Supplementary Table 3). The findings for the expressive suppression ERQ subscale indicated a significant effect for interaction (Supplementary Table 3). For the harsh and inconsistent discipline PPI subscale and the praise and positive incentives PPI subscale, significant interaction effects were found (Supplementary Table 3).

### Predictors of Child Wellbeing

As described earlier (Ulfsdotter et al, 2014), significant time and interaction effects were found for CW. Parents in the intervention group reported higher CW than the control group and stated an overall greater change across the follow-up period (Ulfsdotter et al, 2014). To examine whether changes in ERQ and PPI during the intervention predicted changes in CW from baseline to the 6-month follow-up, a linear regression analysis was conducted within the intervention group (Table 3). For each predictor variable, change variables were produced by subtracting the post-intervention scores from the pre-intervention scores. The outcome variable, change in CW, was created by subtracting the CW scores at 6 months from the baseline scores. Consequently, a larger negative change in the CW value shows that participants’ reported scores increased to a greater extent over the measurement periods. A significant but small correlation was obtained for changes in both cognitive reappraisal and harsh and inconsistent discipline to changes in CW (*r* = 0.122, *p* < 0.05; *r* = −0.208, *p* < 0.005). Only harsh and inconsistent discipline predicted a significant change in CW (*β* = −0.21, *t* = −2.975, *p* < 0.005), explaining 4.3% of the variance. A decrease in the perceived use of negative practices during the intervention period partly predicted an increase in parents’ reported CW at the 6-month follow-up. No other significantly predicted changes in CW were demonstrated for the variables parental cognitive reappraisal, expressive suppression, or praise and positive incentives. When PSE was added as a predictor together with the secondary outcomes, only PSE predicted a significant change in CW (*β* = −0.34, *t* = −4.30, *p* < 0.001), explaining 8.5% of the variance (Supplementary Table 4).

**Table 3:** Linear regression estimates for the predictive relationship between changes in ERQ and PPI scores from pre- to post-measurement and changes in CW over the 6-month follow-up period

**Predictor variables**	***N***	**∆*M* (SD)**	**Unstandardized coefficients**		**Coefficients**	***t***	***R***^**2**^
			***B***	***SE***	***β***		
**ERQ subscales**							
Cognitive reappraisal	215	−0.525 (0.986)	1.375	0.765	0.77	0.122	0.015
Expressive suppression	215	−0.152 (0.903)	−0.970	0.833	−0.08	−1.164	0.006
**PPI subscales**							
Harsh and inconsistent discipline	197	3.058 (7.888)	−0.293	0.098	−0.21	−2.975**	0.043
Praise and positive incentives	203	−0.934 (6.008)	0.078	0.129	0.04	0.603	0.002

*ERQ* Emotion Regulation Questionnaire, *PPI* Parenting Practices Interview, *Mean ∆* change calculated as the baseline score minus the post-intervention score

**p < 0.01 (95% confidence interval)

## Discussion

The present study investigated the effects of a Swedish health-promoting universal parenting program on parents’ emotion regulation and parenting practices over a 6-month follow-up period. Parents in the intervention group reported a significant increase in the use of cognitive reappraisal strategies from baseline to 6 months compared to no change in the control group. This corresponded to a small effect size (*d* = 0.28). Compared with parents in the control group, parents in the ACF group did not appear to significantly change their use of ERQ expressive suppression strategies, as both groups impaired over the study period. Regarding parenting practices, an interaction effect was found in both PPI harsh and inconsistent discipline and PPI praise and positive incentives. Parents in both the intervention and the control groups demonstrated a significant decrease in the use of negative, harsh, parenting practices, which was greater in the intervention group. Parents participating in ACF perceived a greater increase in positive practices from baseline to the post-intervention and to the 6-month follow-up than did those in the control group. Associations were found between changes in parental self-efficacy and the effects of cognitive reappraisal strategies in the control group and between changes in parental self-efficacy and negative parenting practices in the intervention group. Child gender moderated positive parenting practices with parents to girls in the intervention group, as indicated by a greater increase in praise and positive incentives than for parents to girls in the comparison group. Study completer analyses and analyses per protocol supported the findings from the GLMM analyses. Regarding predictors, only negative parenting practices showed a predictive relationship with changes in CW at follow-up. However, the predictive relationship of negative parenting practices disappeared when parental self-efficacy was added to the analysis, suggesting that increased parental self-efficacy was associated with improvement in CW at 6 months.

Previous studies evaluating universally offered group-based parenting programs have presented similar findings related to parental outcomes (Salari & Enebrink, 2018). Improved parental emotion regulation has been found in Tuning in to Kids, although comparisons to our study are difficult, as different measures have been used (Wilson et al., 2012). An increase in positive parenting practices has been reported regarding Tuning in to Kids (Wilson et al., 2012) and in the evaluation of the Incredible Years program when used universally (Reedtz et al., 2011). Likewise, other RCTs provide evidence for the advantages of universal programs for reducing parents’ use of harsh, dysfunctional, and inconsistent discipline (Hahlweg et al., 2010; Heinrichs et al., 2014; Reedtz et al., 2011). In contrast, other studies reported no program effect on negative parenting outcomes (Simkiss et al., 2013; Wilson et al., 2012). To our knowledge, research on how intervention-produced changes in parental self-efficacy may mediate changes in other parental outcomes in universal programs is lacking. However, greater effects of parental self-efficacy in a universal program have been reported in relation to more severe initial child problems (Sanders et al 2014). The inclusion of more complex modeling of outcomes could contribute to the continuous delivery and development of universal programs. Knowledge about moderators in universally delivered programs seems to be mixed with respect to ACF (Enebrink et al., 2015; Hahlweg et al., 2010; Sanders et al., 2014; Ulfsdotter et al., 2014). Moderators for parental emotion regulation have not been included in earlier studies of universal programs, while parenting practices have been evaluated in single- and two-parent families with no changes for single mothers (Hahlweg et al., 2010). The current findings suggest that participating in the ACF program enhanced parents’ cognitive reappraisal and parenting practices strategies, but additional research is needed to better understand how changes in these variables interact with each other (i.e., does intervention-produced change in cognitive reappraisal mediate changes in parenting practices?) and to what extent they influence child outcomes. Furthering our understanding of the causal effects of early-life parent–child interactions on child wellbeing later in life will help inform the future design of effective health-promoting UPPs.

Common for this and other studies of universal parent programs are reports of small or moderate effect sizes (Enebrink et al., 2015; Hahlweg et al., 2010; Sanders et al., 2014; Ulfsdotter et al., 2014; Wilson et al., 2012), and it has been highlighted that universal programs may have lower effect sizes, as there is less room for improvement compared to indicated or selective programs (Tanner-Smith et al., 2018). Furthermore, it has been suggested to consider the variation within universal programs where some individuals may gain and others not.

In this study, the PSE was the only parental outcome found to predict CW, which is in line with findings from a pilot study of the ACF (Enebrink et al., 2015). Child development is a complex and multidimensional process, and the environment has been identified by several theorists as a primary mechanism in a child’s development (Krishnan, 2010). This perspective of child development can be used to explain why the current results revealed that only one variable predicted a change in CW to a limited extent. Theories such as Bronfenbrenner’s bioecological model of human development state that multiple factors related to the child (i.e., temperament), other parental factors (i.e., parental stress), family-based factors (i.e., family conflict, lower socioeconomic status), and the external environment (i.e., neighborhood safety, school environment) interact to predict changes in CW (Krishnan, 2010). Overall, this study provides evidence that parenting skills training benefits parents not only in at-risk families but also in the general population. The ACF may not differ from other universal or selective/indicated programs with components from social learning and attachment theory, but there might be variations in how the programs are delivered. Regarding the number of sessions, the ACF is a short program compared to the indicated programs, such as Comet or Connect. The format is group-based without other individual contacts or teacher meetings, which could be compared to a variant of the universal Triple P, which combines four group sessions with the same amount of individual phone contacts (Sanders et al., 2014), or with Video-feedback Intervention to Promote Positive Parenting (VIPP), which places emphasis on individual home visits (Juffer et al., 2018).

### Strengths and Limitations

Using a positive outcome measure of CW is a significant strength of this study. Most universal parenting programs are risk-reduction interventions, which may be disadvantageous because evaluations may find small or no effects since the proportion of children with behavioral problems is likely to be lower in the general population (Salari & Enebrink, 2018). Our findings lend to a gap in research regarding universal programs’ potential health-promoting benefits for families and broader population health outcomes. The use of an RCT method and follow-up measure is a primary strength for adequately examining program effectiveness and whether program effects are maintained over time and is an important step forward for future research on health-promoting parenting programs. As the results show that some effects declined after the intervention, a longer follow-up RCT would be beneficial.

One of the limitations is that the study only used parent-reported measures. An earlier study showed differences between clinical observation measures and parent-reported measures of mother–child interaction and parent behavior (Hahlweg et al., 2010). The measures used in this study were, however, reported to be reliable in similar populations, but the evaluation could be further strengthened by using multiple reporters or various data collection methods. Another limitation is that the effects of having only one of two parents participating in the program were not investigated. Since almost 40% of participants reported that they participated in ACF without their partner, the effects of the program on CW may be underestimated. Additionally, participant attrition may result in selection bias and thus threaten the study’s internal validity. Lower participation rates of parents born outside of Sweden were found at both post- and follow-up measurements. Non-participants were also less educated, had younger children, and stated a lower household income compared to participants. Sociodemographic differences among study participants are a serious limitation in this context, as the intention of universal programs is to increase inclusion and participation to reduce health gaps in the general population. The risk of bias from non-random attrition and the overestimation of intervention effectiveness may be reduced in this study due to the use of ITT analyses. The data in this study were collected from 2012 to 2013, and an evaluation of the ACF with more recent information may add to our findings.

### Future Directions

The present research provides support for the use of a health-promoting parenting program in the general population. The findings demonstrated short- and medium-term effects of the ACF program on improving parental outcomes, with some effects being sustained over time. Continuation of change, especially in an RCT, may be a more significant demonstration of intervention effectiveness under real-world conditions and is critical when establishing an intervention as an effective public health strategy for policymakers.

Nevertheless, more research is required to address the limitations of this study, validate the effectiveness of the ACF program, and establish universal health-promoting interventions as feasible public health strategies for strengthening child wellbeing at the societal level. Further studies might include longer follow-ups to identify the mechanisms of change in such interventions. This study revealed only one aspect that predicted CW, which accounted for less than 9% of the variance, which is why additional research is required to understand the variance of change to a greater extent. Moreover, there will always be obstacles at the structural and operational levels for implementing and sustaining a universal program, such as a lack of resources. An evaluation of the implementation of the ACF would support further program delivery, which could strengthen parents’ participation. Services that are easily and equally available to the whole population are necessary for an effective public health approach to parenting. Nevertheless, it has been reported that parents’ limited access to UPPs is a common barrier to the utilization and implementation of this strategy (Hahlweg et al., 2010). While delivering different approaches and program durations may moderate issues concerning accessibility, it should not be at the expense of program effectiveness. As UPPs developed to promote positive CW are a novel area of study, further studies are required to inform best practices for policymakers.

## Conclusion

The ACF program seems to strengthen parenting practices and parental emotion reappraisal strategies. This study strengthens the evidence for the predictive effect of parental self-efficacy on increasing CW in the framework of a universal health-promoting program. Since the reach and access of interventions are fundamental to universally delivered programs, further implementation evaluations may support program design and participation and ultimately improve the possibility of successfully promoting health at a societal level.

### Supplementary Information

Below is the link to the electronic supplementary material.

Supplementary file1 (DOCX 33 KB)

## Data Availability

Ethical guidelines in Sweden does not allow for public repository archiving of data included in this study. Researchers with ethical permission under Swedish law can contact corresponding author to get access to de-identified data.
