# Supplementary material for: “All Children in Focus”: Effects of a Universal Parenting Program at a 6-Month Follow-Up in a Randomized Controlled Trial in Sweden
Source: Prev Sci. 2024 May 15;25(4):673–84. doi: 10.1007/s11121-024-01681-y (PMC11111508; doi:10.1007/s11121-024-01681-y)
Supplement: Supplementary file 1 — Supplementary file1 (DOCX 33 KB) [file 11121_2024_1681_MOESM1_ESM.docx]

**Supplementary Table 1**

*Mean score (standard error) at each measurement time point with GLMM-based repeated measures group X time interaction x Increase in PSE at 2 weeks post-intervention effect of the outcome measures for participants in the intervention group (n=317) the control (n=295) group*

| **Outcome measures** | | **T1**^a^ **Increase PSE** | **No Increase PSE** | **T2**^b^ **Increase PSE** | **No Increase PSE** | **T3**^c^ **Increase PSE** | **No Increase PSE** | **GLMM** |
| --- | --- | --- | --- | --- | --- | --- | --- | --- |
|  |  | *M (SE)* | *M (SE)* | *M (SE)* | *M (SE)* | *M (SE)* | *M (SE)* |  |
|  | **ERQ. Cognitive Reappraisal** | | | |  |  |  |  |
| Intervention | | 4.39 (0.07) | 4.61 (0.10) | 4.92 (0.07) | 4.77 (0.11) | 4.77 (0.08) | 4.70 (0.12) | *F*(5,1625)=3.72, *p* =.002 |
| Control | | 4.60 (0.08) | 4.59 (0.09) | 4.75 (0.08) | 4.45 (0.10) | 4.67 (0.08) | 4.49 (0.10) |  |
|  | **ERQ. Expressive Suppression** | | | |  |  |  |  |
| Intervention | | 2.79 (0.08) | 2.82 (0.11) | 2.91 (0.08) | 2.75 (0.12) | 2.89 (0.08) | 3.03 (0.13) | *F*(5,1624)=1.82, *p* =.107 |
| Control | | 2.92 (0.09) | 2.06 (0.11) | 3.10 (0.09) | 2.80 (0.11) | 2.97 (0.09) | 2.84 (0.11) |  |
|  | **PPI. Harsh & Inconsistent Discipline** | | | |  |  |  |  |
| Intervention | | 44.53 (0.71) | 41.41 (0.91) | 40.50 (0.65) | 41.65 (0.99) | 41.33 (0.68) | 40.78 (1.02) | *F*(5,1580)=4.61, *p* <.001 |
| Control | | 44.10 (0.75) | 43.08 (0.87) | 42.55 (0.73) | 42.70 (0.89) | 41.99 (0.74) | 42.30 (0.89) |  |
|  | **PPI. Praise & Positive Incentives** | | | |  |  |  |  |
| Intervention | | 42.24 (0.60) | 39.56 (0.81) | 43.46 (0.60) | 39.44 (0.88) | 43.59 (0.61) | 40.35 (0.92) | *F*(5,1594)=2.19, *p* =.053 |
| Control | | 42.60 (0.64) | 41.37 (0.75) | 41.70 (0.64) | 41.46 (0.78) | 42.64 (0.66) | 40.57 (0.79) |  |

*a. T1= pre-intervention b. T2= 2 weeks post-intervention c. T3= 6 months post-baseline d. ERQ= Emotion Regulation Questionnaire e. PPI= Parenting Practices Interview,
*p<0.05, **p<0.01, *** p<0.001 (95% Confidence Interval)*

**Supplementary Table 2**

*Mean score (standard error) at each measurement time point with ANOVA-based repeated measures group X time interaction effect of the outcome measures by intervention and control group including non-attenders in the intervention group answering questionnaires*

| **Outcome  Measures** | **T1**^a^ | **T2**^b^ | **T3**^c^ | **ANOVA** | **t-tests** |  | |
| --- | --- | --- | --- | --- | --- | --- | --- |
|  | *M (SE)* | *M (SE)* | *M (SE)* |  | T1 - T2 | T2 - T3 | T1 - T3 |
| **ERQ. Cognitive Reappraisal** | | |  |  |  |  |  |
| Intervention (n=244) | 4.42 (0.07) | 4.92 (0.06) | 4.76 (0.06) | *F*(2,488)=14.77, *p*<.001 | -0.50*** | -0.16* | -0.34*** |
| Control (n=247) | 4.63 (0.07) | 4.65 (0.06) | 4.61 (0.06) |  | -0.02 | 0.05 | 0.03 |
| **ERQ. Expressive Suppression** | | |  |  |  |  |  |
| Intervention (n=244) | 2.77 (0.07) | 2.88 (0.07) | 2.92 (0.07) | *F*(2,488)=0.27, *p* =.765 | -0.11 | -0.04 | -0.15* |
| Control (n=247) | 2.76 (0.07) | 2.89 (0.07) | 2.66 (0.07) |  | -0.12* | 0.03 | -0.10 |
| **PPI. Harsh & Inconsistent Discipline** | | |  |  |  |  |  |
| Intervention (n=223) | 44.83 (0.68) | 41.56 (0.64) | 42.02 (0.62) | *F*(2,456)=4.60, *p* =.011 | 3.27*** | -0.46 | 2.81*** |
| Control (n=236) | 44.46 (9.65) | 43.30 (0.63) | 42.15 (0.60) |  | 1.17* | 0.60 | 1.77** |
| **PPI. Praise & Positive Incentives** | | |  |  |  |  | |
| Intervention (n=227) | 41.13 (0.58) | 42.15 (0.56) | 42.40 (0.56) | *F*(2,465)=3.23, *p* =.041 | -1.02* | -0.25 | 1.27** |
| Control (n=241) | 41.67 (0.56) | 41.38 (0.54) | 42.69 (0.54) |  | 0.29 | -0.25 | .04 |

*a. T1= pre-intervention b. T2= 2 weeks post-intervention c. T3= 6 months post-baseline d. ERQ= Emotion Regulation Questionnaire e. PPI= Parenting Practices Interview,
*p<0.05, **p<0.01, *** p<0.001 (95% Confidence Interval)*

**Supplementary Table 3**

*Mean score (standard error) at each measurement time point with ANOVA-based repeated measures group X time interaction effect of the outcome measures for participants in the intervention group attending 1-4 sessions and the control group*

| **Outcome  Measures** | **T1**^a^ | **T2**^b^ | **T3**^c^ | **ANOVA** | ***t*-tests** | |  | |
| --- | --- | --- | --- | --- | --- | --- | --- | --- |
|  | *M (SE)* | *M (SE)* | *M (SE)* |  | T1 - T2 | | T2 - T3 | T1 - T3 |
| **ERQ. Cognitive Reappraisal** | | |  |  |  | |  |  |
| Intervention (n=240) | 4.41 (0.07) | 4.92 (0.06) | 4.76 (0.07) | *F*(2,484)=15.11, *p*<.001 | -0.51*** | | 0.16** | -0.35*** |
| Control (247) | 4.63 (0.07) | 4.65 (0.06) | 4.60 (0.06) |  | -0.02 | | 0.05 | 0.03 |
| **ERQ. Expressive Suppression** | | |  |  |  | |  |  |
| Intervention (n=240) | 2.75 (0.07) | 2.86 (0.07) | 2.90 (0.08) | *F*(2,484)=0.33, *p* =.723 | -0.11 | | -0.05 | -0.16* |
| Control (n=247) | 2.76 (0.07) | 2.89 (0.07) | 2.86 (0.07) |  | -0.12* | | 0.03 | -0.10 |
| **PPI. Harsh & Inconsistent Discipline** | | |  |  |  | |  |  |
| Intervention (n=219) | 44.92 (0.68) | 41.65 (0.65) | 42.10 (0.62) | *F*(2,452)=4.55, *p* =.013 | 3.28*** | | -0.46 | 2.82*** |
| Control (n=236) | 44.46 (0.66) | 43.30 (0.63) | 42.69 (0.60) |  | 1.17* | | 0.60 | 1.77* |
| **PPI. Praise & Positive Incentives** | | |  |  |  |  |  | |
| Intervention (n=223) | 41.01 (0.58) | 42.08 (0.56) | 42.32 (0.56) | *F*(2,461)=3.43, *p* =.033 | -1.07** | | -0.24 | -1.31** |
| Control (n=241) | 41.67 (0.56) | 41.38 (0.54) | 41.63 (0.54) |  | 0.29 | | -0.25 | 0.04 |

*a. T1= pre-intervention b. T2= 2 weeks post-intervention c. T3= 6 months post-baseline d. ERQ= Emotion Regulation Questionnaire e. PPI= Parenting Practices Interview,
*p<0.05, **p<0.01, *** p<0.001 (95% Confidence Interval)*

**Supplementary Table 4**

*Linear regression estimates for the predictive relationship between changes in ERQ, PPI and PSE scores from pre- to post-measurement and change in CW over the 6-month follow-up period.*

| **Predictor Variables** | **N** | **∆M (SD)** | **Unstandardized Coefficients** | | **Coefficients** | ***t*** | **R^2^** |
| --- | --- | --- | --- | --- | --- | --- | --- |
|  |  |  | B | *SE* | *ß* |  |  |
| **ERQ Subscales** |  |  |  |  |  |  |  |
| Cognitive Reappraisal | 215 | -.0.508 (0.999) | 0.228 | 0.791 | 0.021 | 0.289 | 0.016 |
| Expressive Suppression | 215 | -0.132 (0.927) | -1.277 | 0.822 | -0.107 | -1.553 | 0.007 |
| **PPI Subscales** |  |  |  |  |  |  |  |
| Harsh & Inconsistent Discipline | 197 | 3.018 (7.815) | -0.089 | 0.108 | -0.063 | -0.826 | 0.035 |
| Praise & Positive Incentives | 203 | -0.956 (5.971) | -0.116 | 0.133 | 0.063 | -0.872 | 0.005 |
| **Parental Self-Efficacy** | 213 | -24.458 (39.922) | 0.093 | 0.022 | 0.335 | 4.298*** | 0.085 |

*Note. ERQ= Emotion Regulation Questionnaire, PPI= Parenting Practices Interview,* Mean **∆** *= change calculated as baseline score minus post-intervention score, *p<0.05, **p<0.01, *** p<0.001 (95% Confidence Interval)*
